# Elevator block brake structural optimization design based on an approximate model

**DOI:** 10.1371/journal.pone.0296753

**Published:** 2024-03-28

**Authors:** Haijian Wang, Chengwen Yu, Xishan Zhu, Liu Jian, Congcong Lu, Xiaoguang Pan

**Affiliations:** 1 School of Mechanical and Electrical Engineering, Guilin University of Electronic Technology, Guilin Guangxi, Guilin, China; 2 Guangxi Institute of Special Equipment Inspection and Research, Guilin Guangxi, Guilin, China; Ningbo University, CHINA

## Abstract

An Aquila optimizer-back propagation (AO-BP) neural network was used to establish an approximate model of the relationship between the design variables and the optimization objective to improve elevator block brake capabilities and achieve a lightweight brake design. Subsequently, the constraint conditions and objective functions were determined. Moreover, the multi-objective genetic algorithm optimized the structural block brake design. Finally, the effectiveness of the optimization results was verified using simulation experiments. The results demonstrate that the maximum temperature of the optimized brake wheel during emergency braking was 222.09°C, which is 36.71°C lower than that of 258.8°C before optimization, with a change rate of 14.2%. The maximum equivalent stress after optimization was 246.89 MPa, 28.87 MPa lower than that of 275.66 MPa before optimization, with a change rate of 10.5%. In addition, the brake wheel mass was reduced from 58.85 kg to 52.40 kg, and the thermal fatigue life at the maximum equivalent stress increased from 64 times before optimization to 94 times after optimization.

## 1 Introduction

Emergency elevator block braking is a complex thermal-structural coupling problem. During emergency braking, a significant amount of frictional heat causes a sharp increase in the brake temperature. Excessive temperature decreases the friction torque between the brake wheel and shoe, contributing to thermal cracks on the brake wheel surface, which critically affects the elevator braking ability. Therefore, researching the elevator brake thermal-structural phenomenon during emergency braking to enable the development of a structural optimization brake design to improve the braking capability and ensure safe and stable elevator operation is crucial.

Intelligent algorithms, which stem from integrating computer technology and numerical analysis, have become a research hotspot to solve structural optimization problems, generally achieved by simulating natural phenomena or physical processes [1]. Dhiman proposes a selfish herd optimization algorithm for multi-objective problem optimization, tests it on 24 benchmark functions, and compares it with six recently developed meta-heuristic algorithms. The results show the applicability of this method to practical problems [2]. Kaveh proposed a Lion Pride optimization algorithm to solve the optimal design problem. The effectiveness of the proposed method is verified using 15 mathematical examples and two benchmark structural design problems [3]. Gomes studied truss structures with multiple frequency constraints while using particle swarm optimization (PSO) to optimize the structure’s layout and size [4]. Miguel combined the firefly algorithm (FA) and harmonious search strategy to optimize the size of the multi-frequency constrained structure [5]. Chen proposed a hybrid optimization method by embedding local search operators into the framework of genetic algorithms based on rigorously derived optimality criteria, making the hybrid method suitable for targets involving many structures [6]. Although many scholars have studied intelligent algorithms in structural optimization problems, and the algorithms have certain global search and analysis capabilities, there are also many shortcomings, such as algorithm instability, long iteration duration, and difficulty in solving complex problems. As a new intelligent optimization algorithm proposed in 2021, the Aquila Optimization algorithm (AO) has obvious advantages, including multiple exploration and development strategies, strong optimization ability, fast convergence speed, and an algorithm that is not premature [7]. Serdar proposed an improved, enhanced AO algorithm, which uses an improved opposition learning mechanism (OBL) and Nelder-Mead simplex search method, and applied it to the design of the PID plus second derivative controller (PIDD2) in automatic voltage regulator systems [8]. In the optimization study of the air-fuel ratio system, Davut proposed an improved Aquila optimizer, and the results show that the algorithm has better robustness and stability than other advanced algorithms and also has better running time than other advanced algorithms [9].

Based on advanced algorithms, many scholars have researched proxy models for structural optimization. Liang used Abaqus to numerically simulate the dynamic process of the lifting mechanism of a mine elevator under emergency braking conditions [10]. Based on the finite element simulation results, the Kriging network model between the brake optimization parameters and optimization objectives was established using the Isight software. The structural parameters of the brake under optimal temperature conditions were obtained by combining the adaptive security algorithm and nonlinear programming by Quadratic Lagrange (NLPQL). Cai obtained a disc brake wear model by considering disc brake friction and wear performance, studied the dynamic changes in the wear characteristics of the disc during the entire braking process, analyzed the sensitivity of design parameters and optimization objectives, proposed a cuckoo intelligent optimization algorithm to optimize the approximate model, and completed an optimal brake block structure design [11]. Aiming at nonlinear industrial robot systems, Zhang proposed a hybrid learning algorithm for training radial basis function networks by combining a clustering learning algorithm and an orthogonal least squares learning algorithm demonstrating the method’s high proficiency and reliability through examples [12]. Tang proposed an equivalent deterministic method, which decoupled the double-loop nested optimization into an iterative solution process after establishing an uncertain design optimization model. After significant research, the agent model method has proved reliable and effective in optimizing structural design [13].

Based on the AO-BP neural network, this study establishes an approximate model of the relationship between design variables and optimization objectives, analyzing the prediction accuracy of the approximate model constructed by the AO-BP neural network. Then, the mathematical model of block brake structure optimization is established with the brake wheel’s mass and thermal fatigue life as optimization objectives and the maximum thermal stress and maximum temperature during the emergency braking process as inequality constraints. The Pareto solution is optimized by combining a multi-objective genetic algorithm, and finally, the Pareto solution is substituted back into the finite element simulation model to verify the accuracy of the approximate model output values. Therefore, our model provides an effective way to optimize the design of the brake structure and prolong the service life of the brake.

## 2 Block brake thermal-structural coupling analysis

**Fig 1:**
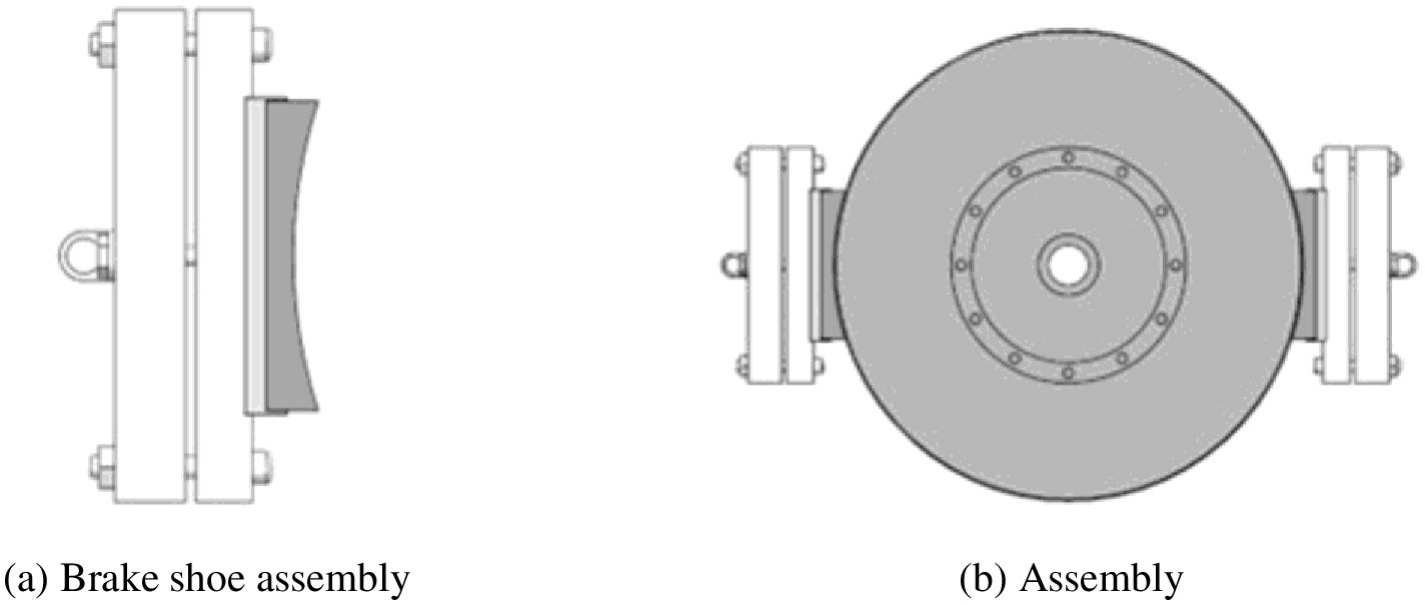
Elevator block brake 3D model. (a) Brake shoe assembly, (b) Assembly.

According to the structural parameters of a certain type of elevator block brake, a three-dimensional (3D) brake model was established using SolidWorks software, as shown in Fig 1. Subsequently, the 3D model was imported into the ANSYS finite element analysis software package, and material setting and mesh division were conducted. The brake wheel is made of HT250 with a density of 7220 kg/m3 and a Poisson ratio of 0.3. The brake shoe is made of resin-based composite material with a density of 1550 kg/m3 and a Poisson ratio of 0.38. The friction coefficient between the brake wheel and brake shoe material is 0.38. In this study, the number of units of the brake shoe, the number of grid nodes is 432, the number of grid nodes is 2355, the number of brake wheel units is 2576, and the number of grid nodes is 13524. Accordingly, the total braking time, heat flow distribution coefficient, and convective heat transfer coefficient parameters were solved according to actual working conditions. Boundary conditions, such as the initial environment and displacement constraint, were established to complete the preprocessing of the block brake finite element model.

The initial ambient temperature is 22°C, the outer surface of the brake wheel is set as the target surface, and the inner surface of the brake shoe is set as the contact surface. The emergency braking process of the elevator is a uniform deceleration motion. Firstly, an initial angular velocity is applied to the rotation center of the brake wheel to release only the freedom of rotation around the z-axis of the rotation center of the brake wheel. At the same time, 1.8 MPa pressure is applied to the outer surface of both sides of the brake shoe to constrain the displacement freedom in the x-axis direction of the outer surface of both sides of the brake shoe. The emergency braking process of the elevator uses the mutual friction between the brake shoe assembly and the brake wheel, and the mechanical energy of the elevator system is converted into the thermal energy of the brake and a small part of other energy forms. When defining the convection distribution coefficient, the heat flux of the input brake shoe is 0.1, and that of the input brake wheel is 0.9, which is determined by the material properties of the friction pair. In the emergency braking process of the elevator, forced convection heat transfer occurs between the brake wheel and the surrounding air. In this process, the convective heat transfer coefficient changes with the speed of the brake wheel. The relationship between the convective heat transfer coefficient on the brake wheel surface and time is as follows [14]:

$$h_{c}=130.5-40.38t$$ (1)

In the actual braking process, the non-contact surface of the brake shoe is fixed on the moving plate by riveting, which is in a limited space, so the convective heat transfer coefficient of the brake shoe is considered unchanged in the thermal analysis. In this study, *h*_*c*_ = 5.3W/m^2^°C.

**Fig 2:**
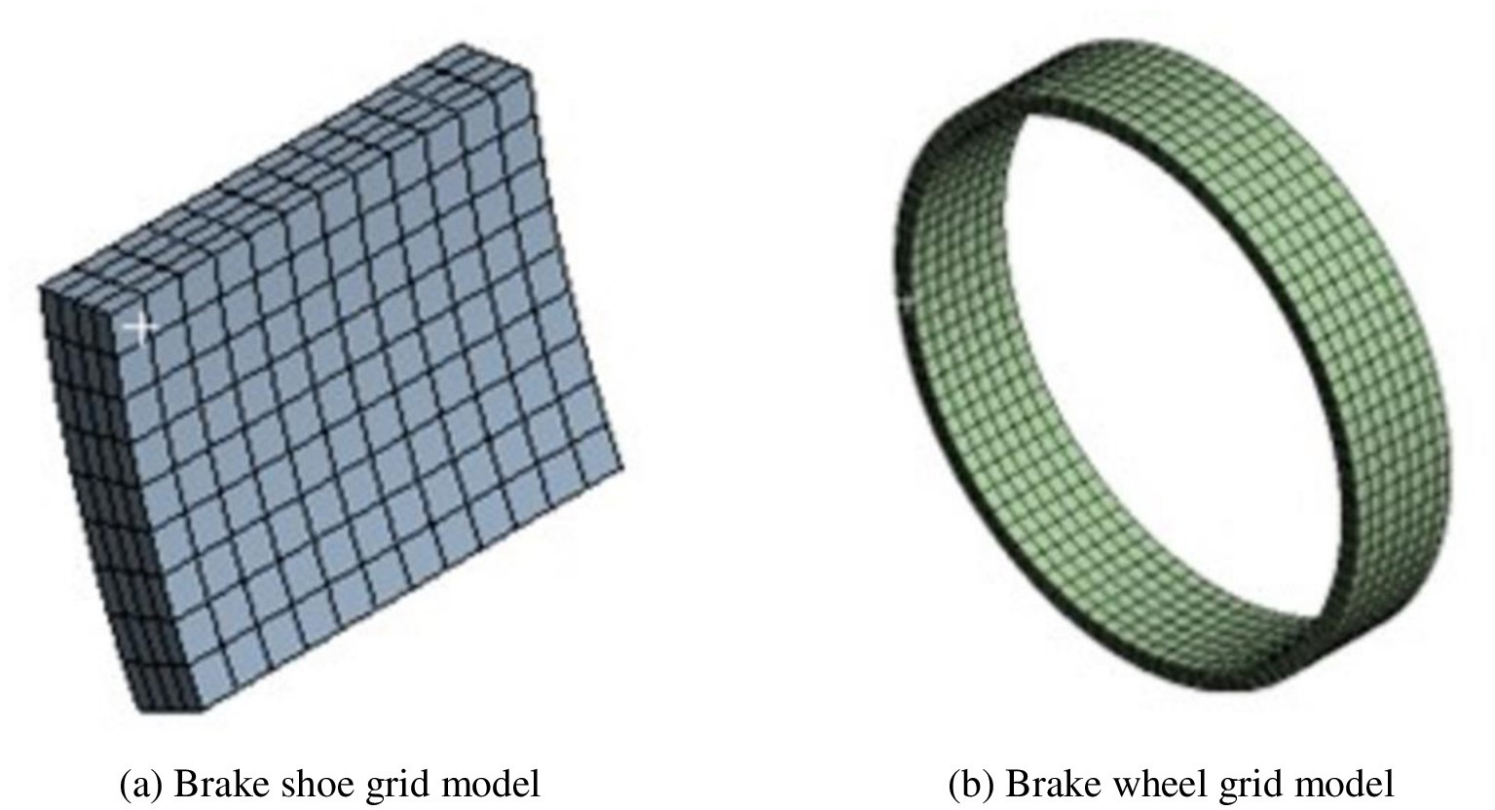
Finite element mesh model of the block brake. (a) Brake shoe grid model, (b) Brake wheel grid model.

Because the brake wheel and shoe almost entirely absorb the heat generated by friction in the actual braking process, only the brake wheel and shoe were considered in the finite element simulation of the emergency block braking process. In this study, to control the scale of the finite element model, irrelevant model details, such as brake wheel holes and shoe chamfers, were removed, and the simplified grid model is shown in Fig 2.

After the finite element model preprocessing and establishing the block brake thermal-structural coupling model, the dynamic change characteristics of the 3D transient temperature and stress fields of the block brake in the emergency braking process were studied based on the aforementioned preset parameters.

**Fig 3:**
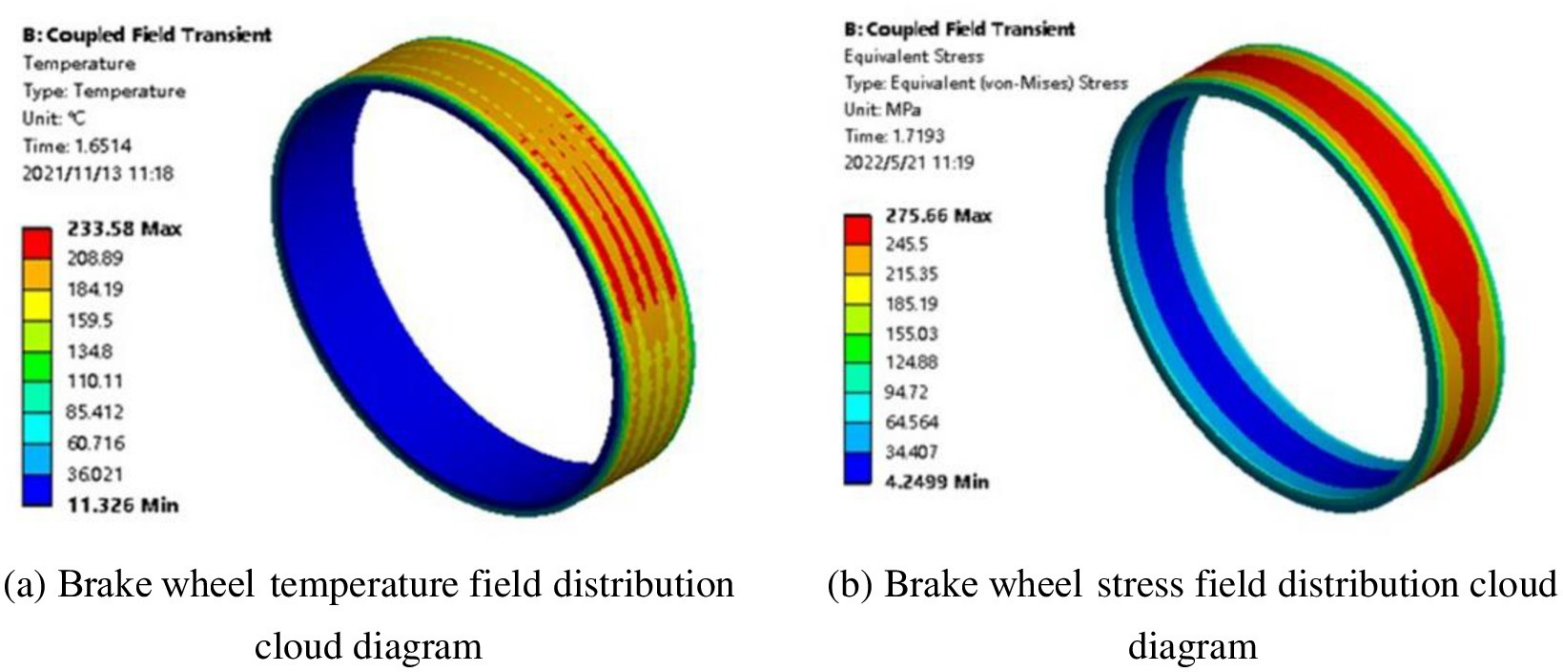
Brake wheel coupling field distribution cloud diagram during the emergency braking process. (a) Brake wheel temperature field distribution cloud diagram, (b) Brake wheel stress field distribution cloud diagram.

Fig 3 shows the brake wheel coupling field distribution cloud diagram during emergency braking. The brake wheel temperature and stress fields were not evenly distributed, and certain gradients existed in the radial, axial, and circumferential directions. When braking time was 1.6514 s, the brake wheel surface temperature reached 233.58°C, and at 1.7193 s, the maximum equivalent stress of the brake wheel was 275.66 MPa. In addition, the brake wheel underwent thermal deformation under an uneven temperature field due to internal and external constraints in the emergency braking process, resulting in significant thermal stress. Because the brake wheel material was HT250 and the yield limit was 250 MPa, the brake wheel maximum equivalent stress exceeded the material yield limit, resulting in plastic deformation of the brake wheel.

**Fig 4:**
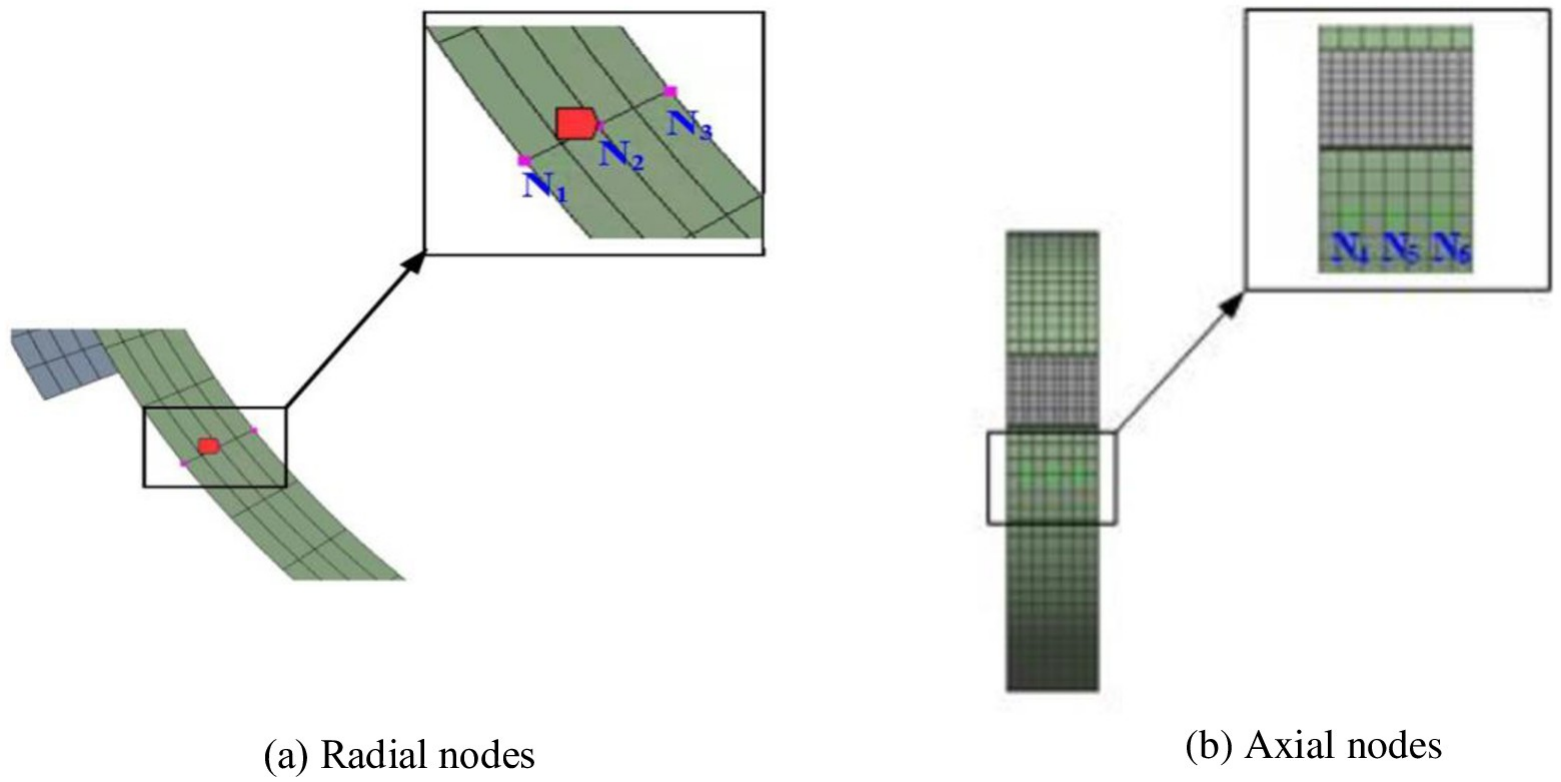
Selected brake wheel radial and axial nodes. (a) Radial nodes, (b) Axial nodes.

A set of brake wheel profile radial nodes N1, N2, and N3 and surface axial nodes N4, N5, and N6 were selected to further analyze the brake wheel temperature variation characteristics in all directions. As shown in Fig 4, the equivalent stress change trend of each node selected in the radial or axial direction during the entire braking process was similar to the temperature change trend of each node. The temperature and stress–time history curves also show zigzag changes, and the small zigzag width is narrow in the early braking stage and gradually widens in the middle and late braking stages. The stress gradients of the radial nodes differed significantly, and the axial nodes exhibited no obvious gradients. The reason for the zigzag change in the temperature–time curve is that the brake wheel friction surface is subjected to the alternating action of frictional heat flow and convective heat dissipation, which also confirms that the thermal stress generated by the temperature change is the primary form of brake wheel equivalent stress during the entire braking process.

In this study, to further understand the change law of the coupling field of the brake wheel over time, the brake wheel profile radial nodes N1, N2, and N3 and the surface axial nodes N4, N5, and N6 were selected.

**Fig 5:**
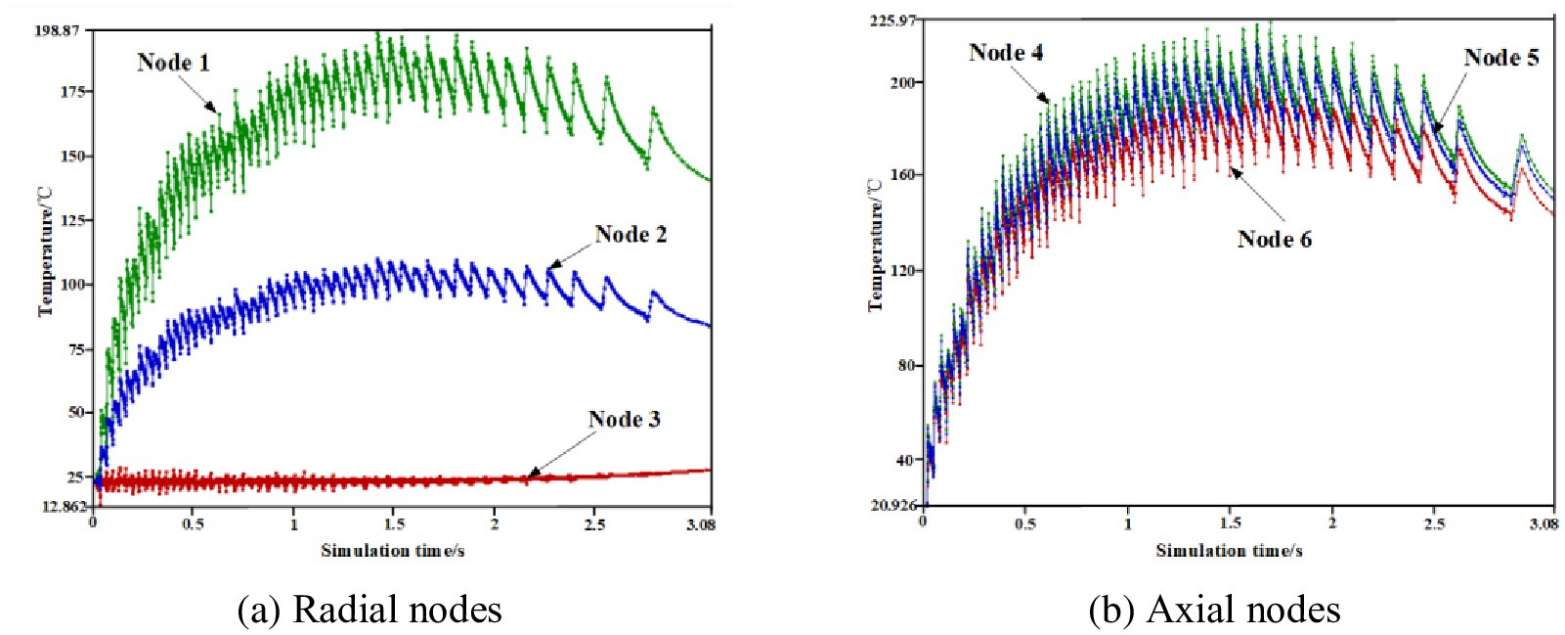
Temperature curve of nodes taken by the brake wheel over time. (a) Radial nodes, (b) Axial nodes.

**Fig 6:**
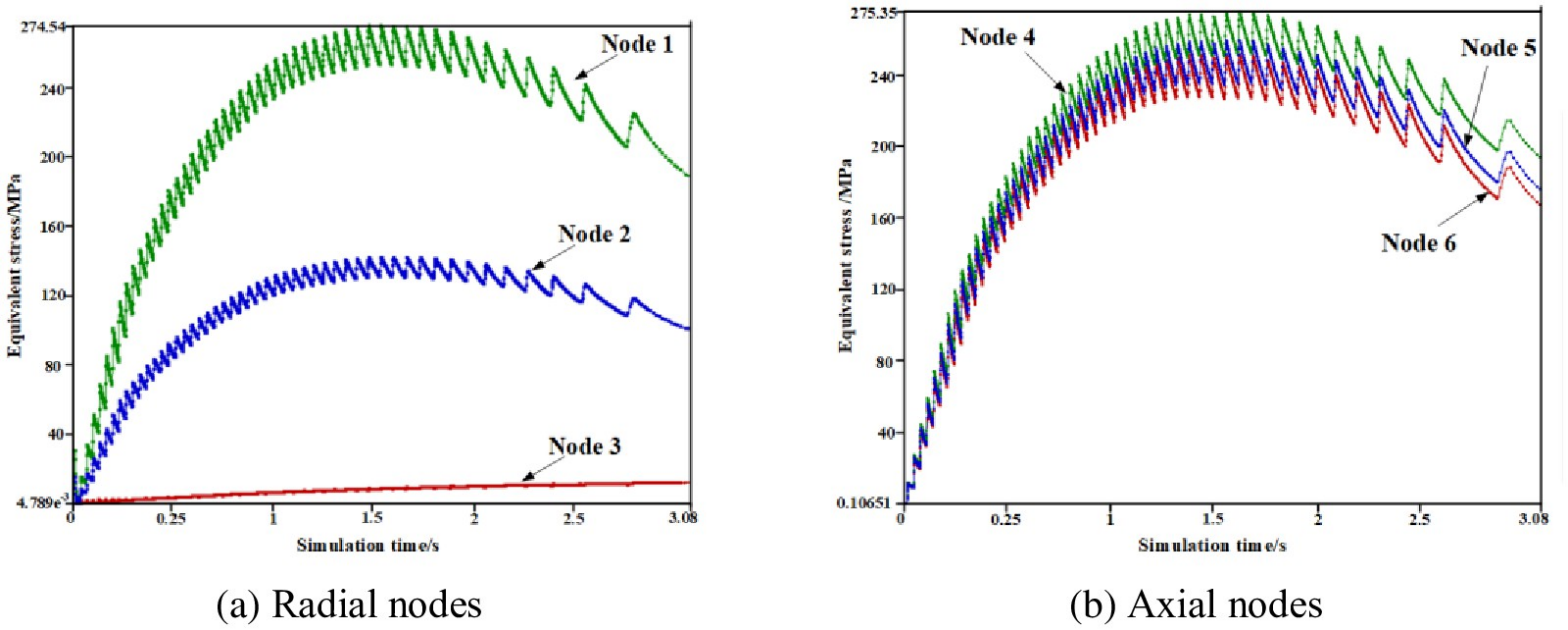
Stress curve of nodes taken by the brake wheel over time. (a) Radial nodes, (b) Axial nodes.

As shown in Fig 5, the temperature of the selected nodes first increased to a peak and then gradually decreased. The temperature–time history curve exhibited a zigzag distribution, and the small zigzag changed from narrow to wide. In addition, the temperature gradient between the radial nodes N1, N2, and N3 was large, and the maximum temperature difference reached 175°C. The temperature difference between the axial nodes N4 and N6 was approximately 30°C. A comparison of Figs 5 and 6 shows that the equivalent stress variation trend of radial and axial brake wheel nodes over time is consistent with that of the temperature. The stress–time history curve presented a zigzag distribution, and the small zigzag changed from narrow to wide. Similarly, a large stress gradient exists between the radial nodes, whereas the temperature gradient of the axial nodes is small. It was also confirmed that the thermal stress generated by the temperature changes during the entire braking process was the primary form of brake wheel equivalent stress.

## 3 Determination of block brake optimization objectives and design variables

### 3.1 Determination of optimization objectives

The selection of optimization objectives varies for block-brake structural optimization. Some researchers selected the natural frequency and warping deformation of the brake as optimization objectives [15], whereas others considered the thermal coupling temperature and stress processes [16]. Since this study mainly investigates the thermal fatigue life of the block brake, the minimum block brake mass and the maximum brake wheel thermal fatigue life were considered objective functions under certain constraints, and a mathematical model for block brake structural optimization was established. Simultaneously, the multi-objective genetic algorithm was combined to achieve the optimal block brake design [17].

### 3.2 Design variable selection

Once the optimization objectives are determined, analyzing the factors affecting them is necessary. Through finite element analysis of the block brake, it was confirmed that temperature and thermal stress are the primary factors affecting braking performance. The change in temperature and thermal stress stems from multiple factors, such as the friction coefficient, the friction pair’s material characteristics, and the structural parameters of the brake. However, the characteristics of the friction material cannot be rapidly optimized. Therefore, some block brake structural parameters were selected as design variables in this study. As one of the optimization objectives, the brake quality is calculated according to its structural characteristics, and the main influencing parameters are the diameter of the brake wheel and the width of the brake shoe (refer to Eq 14). The brake shoe angle determines the contact area between the brake shoe and the brake wheel. The greater the brake angle, the greater the friction area, which is the main way of generating thermal stress in the brake during emergency braking.

The specific selection principles are summarized as follows:

1. Three structural parameters, namely the brake wheel outer radius, brake shoe width, and brake shoe encapsulation angle, were selected as design variables.
2. The design variables’ selection range was reasonable to ensure that no interference occurred between components.
3. The total brake mass should be minimized.

Table 1 shows the range of design variables.

**Table 1:** Range of design variables.

Design variables	Outer radius of the brake wheel *x*_1_ (m)	Width of the brake shoe *x*_2_ (m)	Encapsulation angle of the brake shoe *x*_3_ (°)
Range of variables	0.55–0.65	0.10–0.14	17–19

## 4 Approximate model construction

### 4.1 Approximate model building process

**Fig 7:**
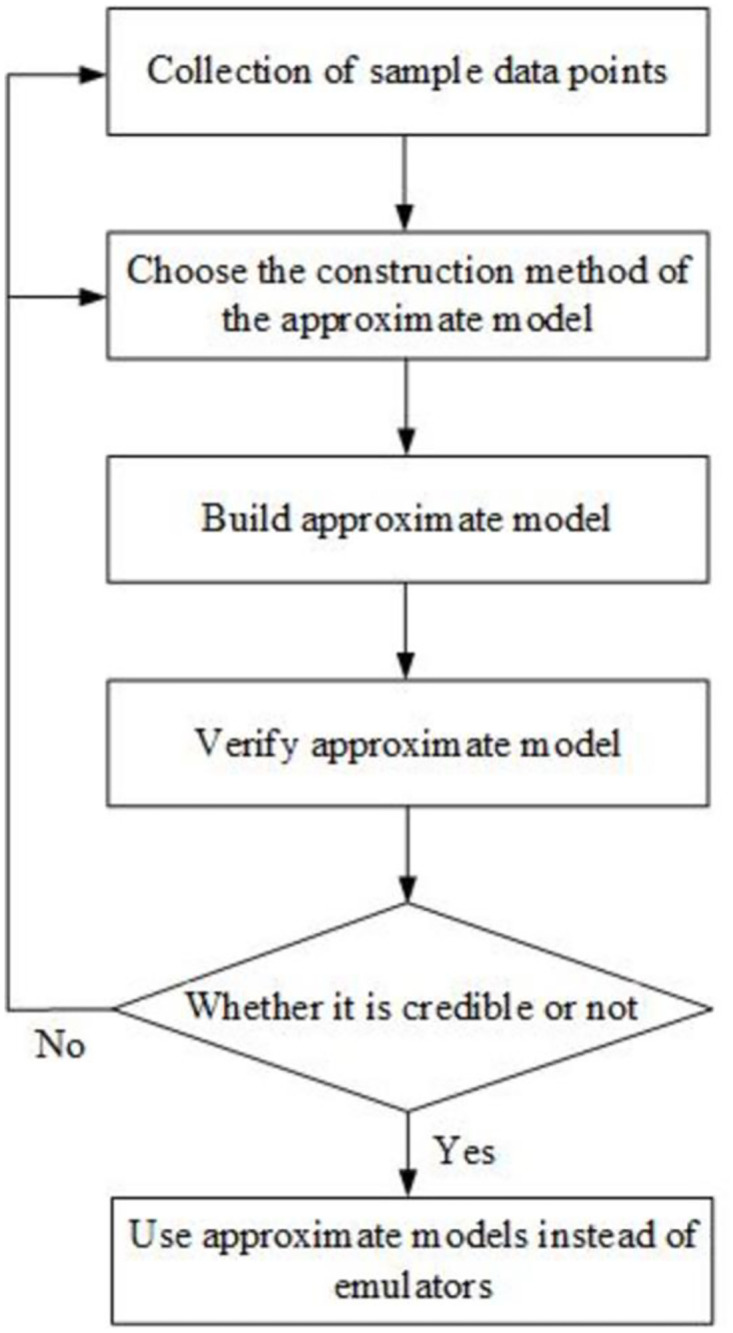
Approximate model construction framework.

Elevator emergency braking is a complex nonlinear problem, and its finite element simulation process involves a lengthy cycle that is difficult to converge [18]. If the finite element brake model is directly improved and the optimized performance is verified using simulation, the time and resource consumption are inestimable. Therefore, researchers have suggested the concept of an approximate model [19]. This approximation model has rapidly developed and is currently considered the mainstream design optimization method. The approximate model instead of the finite element model is widely used to solve the problem of multi-objective structure optimization [20, 21]. It approaches the relationship between design variables and optimization objectives using a high-accuracy mathematical model that avoids high-intensity and high-cost modeling and simulation to a significant extent. Fig 7 shows the approximate model construction framework.

### 4.2 Optimal Latin Hypercube experiment design

**Fig 8:**
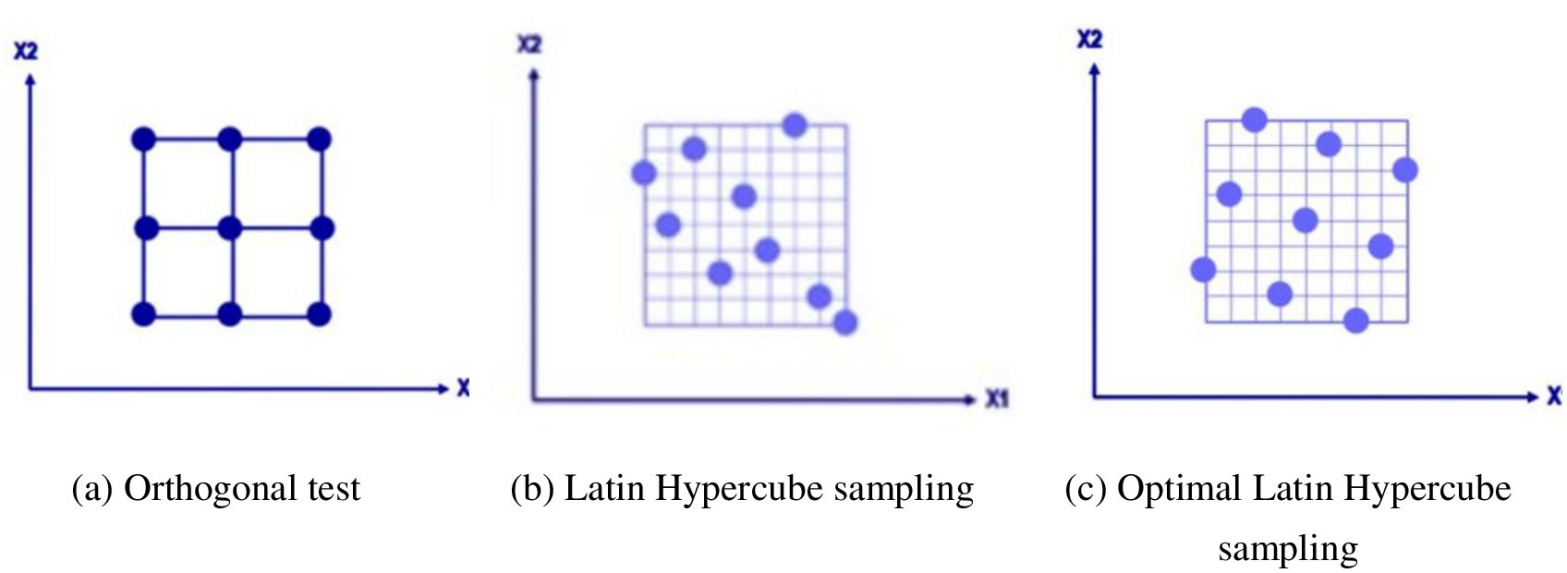
Data sampling methods. (a) Orthogonal test, (b) Latin Hypercube sampling, (c) Optimal Latin Hypercube sampling.

The first step in building an approximate model is to collect sample data. In this study, the Optimal Latin Hypercube sampling method was used to select the sample data points, which is essentially an improvement over the random Latin method [22]. First, each design variable was divided into N identical cells, and subsequently, N values were randomly selected within the N cells. Finally, the N values of each variable were randomly combined with those of other variables. Compared to the orthogonal experimental design, the Optimized Latin Hypercube sampling method maximizes the distribution and stratification of each edge to ensure that the value range of each variable is covered as far as possible. Compared with the traditional Latin hypercube experimental design, this optimizes the order of occurrence of each level so that optimized sample points are more evenly distributed in the interval. Therefore, the sample-selection method based on the Optimal Latin Hypercube experimental design ensured the utmost extent of accuracy and credibility for the approximate model. Fig 8 shows the data sampling methods.

Twenty sample data groups were selected using Optimal Latin Hypercube sampling in the design variable range. The finite element model of the elevator block brake was re-established according to the structural parameter data of each group of design variables, and the simulation was conducted based on identical boundary, contact, and load conditions. The brake wheel maximum temperature *T* and stress *S* during the emergency braking process were obtained and are summarized in Table 2.

**Table 2:** Design variable sampling points and response data.

Serial number	*x*_1_(m)	*x*_2_(m)	*x*_3_(°)	*T*_max_(°C)	*S*_max_(MPa)
1	0.5763	0.1084	18.58	260.99	241.79
2	0.5658	0.1211	17.53	238.37	243.6
3	0.6237	0.1168	17.42	247.81	217.7
4	0.6079	0.1253	17.11	244.48	224.91
5	0.6447	0.1147	17.32	258.59	209.75
6	0.5974	0.1232	18.79	243.28	230.23
7	0.5711	0.1337	18.47	237.79	222.57
8	0.5921	0.1105	18.68	243.02	234.09
9	0.6289	0.1	17.05	217.94	175.15
10	0.6184	0.1021	17.74	265.61	185.32
11	0.5816	0.1042	17.21	246.38	238.97
12	0.5605	0.1358	18.37	220.17	242.45
13	0.6132	0.1379	17.84	236.36	223.39
14	0.6395	0.1316	18.16	247.85	215.08
15	0.5553	0.1189	18.63	243.71	251.58
16	0.5868	0.1274	17.0	251.13	232.48
17	0.65	0.1295	18.26	249.89	187.32
18	0.55	0.1063	18.89	262.03	253.37
19	0.6026	0.14	17.95	274.87	242.38
20	0.6342	0.1126	19.0	283.87	221.66

### 4.3 Back Propagation (BP) neural network

A BP neural network is a type of error-backpropagation algorithm. The BP neural network has stronger learning, memory ability, and training accuracy than other algorithms. Especially for the parameter optimization design of a mechanical structure, the BP neural network can approach any continuous nonlinear function. The core of the BP algorithm is the gradient descent method, in which a sigmoid function is used as the activation function [23]. The algorithm first sets the weights of neurons in the upper layer and those in the next layer. The input values and weights are multiplied using matrix multiplication to obtain the output value. Typically, the bias is also called the threshold in a BP neural network; therefore, the model adjusts its weights and thresholds through repeated training. When the minimum error is reached or the error is less than a certain value, the network model ceases iteration, and the weights and thresholds are considered the optimal solutions.

The number of neurons in the hidden layer can be obtained using Eq 2:

$$n_{hidden}=3\times n_{input}+1$$ (2)

**Fig 9:**
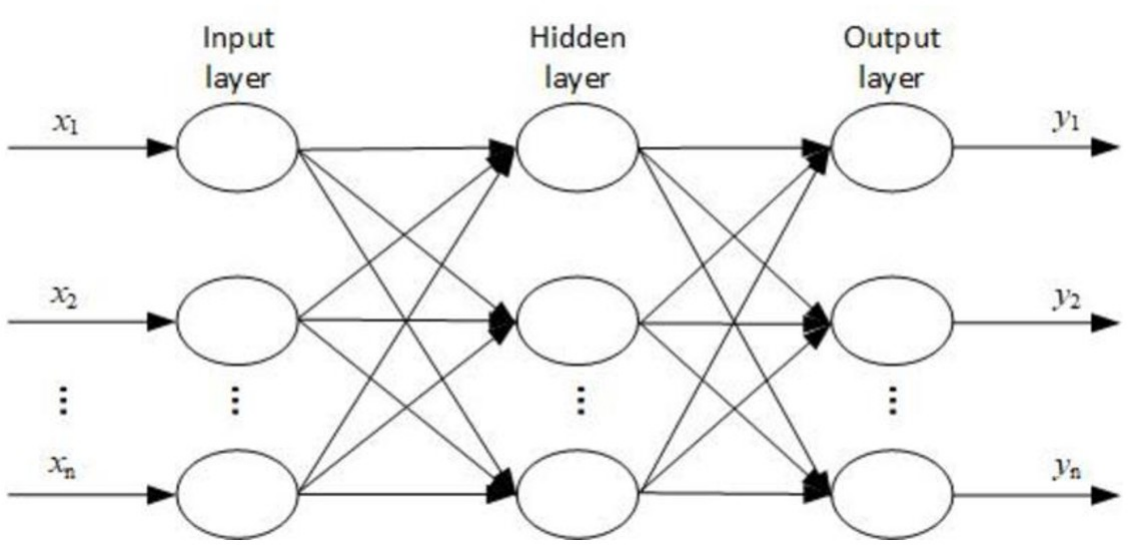
BP neural network structure.

From Fig 9, the brake wheel outer radius *x*_*1*_, brake shoe length *x*_*2*_, and brake shoe encapsulation angle *x*_*3*_ are the design variables. The brake wheel maximum temperature *T*_max_ and maximum stress *S*_*max*_ during the braking process are the optimization objectives; that is, the number of input and output variables of the BP neural network are three and two, respectively. According to Formula 1, the number of hidden layer neurons is 10; therefore, the structure of the constructed BP neural network model is 3-10-2. Simultaneously, the weights and thresholds corresponding to the neural network model were also determined. The input and hidden layer weights were 3 × 10 = 30, and the hidden and output layer weights were 2 × 10 = 20. Simultaneously, the hidden layer had 10 thresholds, and the output layer had two.

The neural network model’s initial weights and thresholds are generally generated by random numbers [-0.5, 0.5]. However, the model corresponding to the weights and thresholds under initialization has low accuracy and slow convergence speed, and it is difficult for the actual output and expected values to meet expectations. The best neural network prediction performance is desirable to improve the approximate model’s accuracy. Therefore, this study used the Aquila optimizer (AO) algorithm to optimize the initial network model weights and thresholds.

### 4.4 BP neural network model optimization based on the AO algorithm

**Fig 10:**
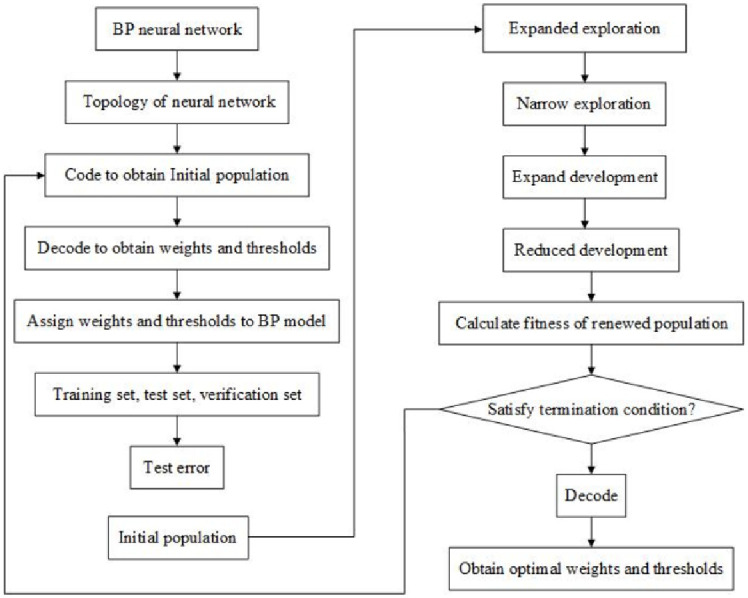
Optimization flow chart of the AO algorithm.

The AO algorithm is a new optimization algorithm proposed by Laith Abualigah in 2021. The algorithm is derived from Aquila’s four methods of hunting ground prey. Compared with traditional heuristic algorithms, such as PSO, ant colony algorithm, and simulated annealing algorithms, it has a more powerful global exploration capability and is more suitable for small sample library optimization. In addition, it is difficult to fall into local optima during the solution process. The AO algorithm optimization principle can be expressed as follows: After initializing the population, the algorithm updates the population fitness value after a series of steps, such as expanding and reducing the search. The fitness value decodes the neural network model after satisfying the termination conditions. Each step is defined in detail as follows: Fig 10 shows the optimization flow chart of the AO algorithm.

1. Initialize the population.
$$X_{ij}=rand\times \left(UB_{j}-LB_{j}\right)+LB_{j},i=1,2,\cdots ,N_{j}=1,2\cdots ,Dim$$ (3)
  Where *rand* is a random value between zero and one, *UB*_*j*_ represents the *j*-th upper bound of a given problem, *LB*_*j*_ represents the *j*-th lower bound of the given problem, and *Dim* represents the search space dimension.
2. Expand search (*X*_*1*_).
$$X_{1}\left(t+1\right)=X_{best}\left(t\right)\times \left(1-\frac{t}{T}\right)+\left(X_{M}\left(t\right)-X_{best}\left(t\right)*rand\right)$$ (4)
  Where *X*(*t*) and *X*(*t+*1) represent individual positions in the *t* and *t+*1 iterations, respectively, *X*_best_(*t*) represents the best individuals obtained by the algorithm up to the *t* iteration, *X*_M_(*t*) represents the average position of the population in the *t* iteration, and *T* represents the maximum number of iterations.
3. Narrow the search (*X*_*2*_).
$$X_{2}\left(t+1\right)=X_{best}\left(t\right)\times Levy\left(D\right)+X_{R}\left(t\right)+\left(y-x\right)*rand$$ (5)
  Where *Levy*(*D*) represents the flight distribution strategy, *D* is the dimensional space size, and *X*_*R*_(*t*) is a random solution within the range [1, N] at the *t* iteration.
4. Expand development (*X*_*3*_).
$$X_{3}\left(t+1\right)=\left(X_{best}\left(t\right)-X_{M}\left(t\right)\right)\times \alpha -rand+\left(\left(UB-LB\right)\times rand+LB\right)\times \delta$$ (6)
  Where *α* and *δ* represent development adjustment parameters and are both 0.1.
5. Narrow development (*X*_*4*_).
$$X_{4}\left(t+1\right)=QF\times X_{best}\left(t\right)-\left(G_{1}\times X\left(t\right)\times rand\right)-G_{2}\times Levy\left(D\right)+rand\times G_{1}$$ (7)
  Where *QF*(*t*) represents the mass function value used to balance the search strategy, *G*_*1*_ represents the various AO movements during the prey tracking process, and *G*_*2*_ represents the linearly decreasing flight slope value of the range [0, 2].

### 4.5 Prediction accuracy analysis of the AO-BP neural network approximation model

The AO algorithm was adopted to optimize the BP neural network model. First, the sample data were normalized, and the training and test sets were divided. In this study, a ratio of 7:3 was used; 14 sample data groups were used for the training set; and six sample data groups were used for the prediction and error analysis of the optimized BP neural network model. The average relative error between the predicted and real values was used as the evaluation criterion to determine model accuracy.

**Fig 11:**
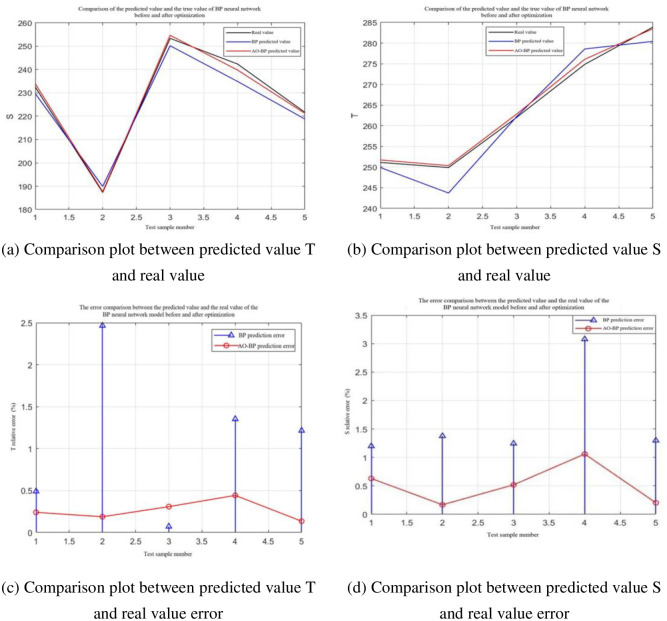
Error comparison between predicted and real BP neural network model values before and after AO optimization. (a) Comparison plot between predicted value T and real value, (b) Comparison plot between predicted value S and real value, (c) Comparison plot between predicted value T and real value error, (d) Comparison plot between predicted value S and real value error.

Fig 11 shows the error comparison between the predicted and real BP neural network model values before and after the AO algorithm optimized the weight and threshold. It can be observed that the output predicted by the non-optimized BP neural network model does not fit the real-value curve. After the weights and thresholds are optimized using the AO algorithm, the output predicted by the AO-BO neural network model fits the real value curves *T* and *S*. As shown in Table 3, the average relative error between the predicted value curve *T* and the actual value was 0.5%. In contrast, the maximum average relative error between the predicted value curve *S* and the actual value was only 1%.

**Table 3:** Error analysis.

Model	Mean absolute error (mae)	Mean square error (mse)	Mean relative error (mre)	Coefficient of determination (R^2^)
BP	3.8	17.6	1.6%	0.97
AO-BP	1.2	2.2	0.5%	0.99

**Fig 12:**
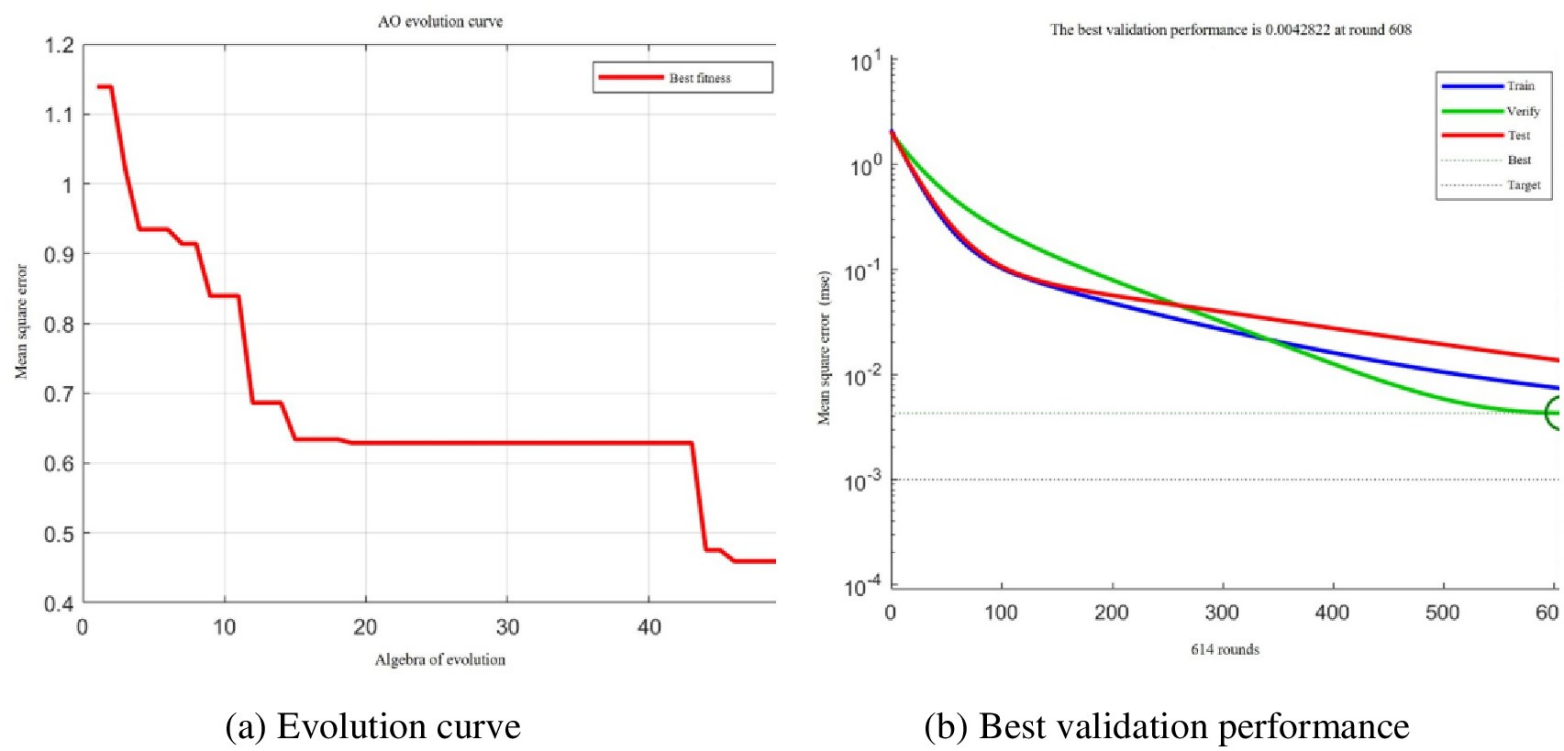
AO-BP iterative process. (a) Evolution curve, (b) Best validation performance.

Fig 12 shows the AO-BP iterative process. After the optimal number of hidden layer nodes reaches eight, the corresponding Euclidean distance error is 1.3172. In addition, the fitness function selection directly affects the AO algorithm convergence rate and whether the optimal solution can be identified. Generally, the fitness function is transformed by the objective function. It can be observed from Fig 10 that when the current iteration number reaches 50, the optimal fitness value thus far is 0.459618. In addition, the AO-BP model reaches an error of 0.0042822 after 608 training iterations.

**Fig 13:**
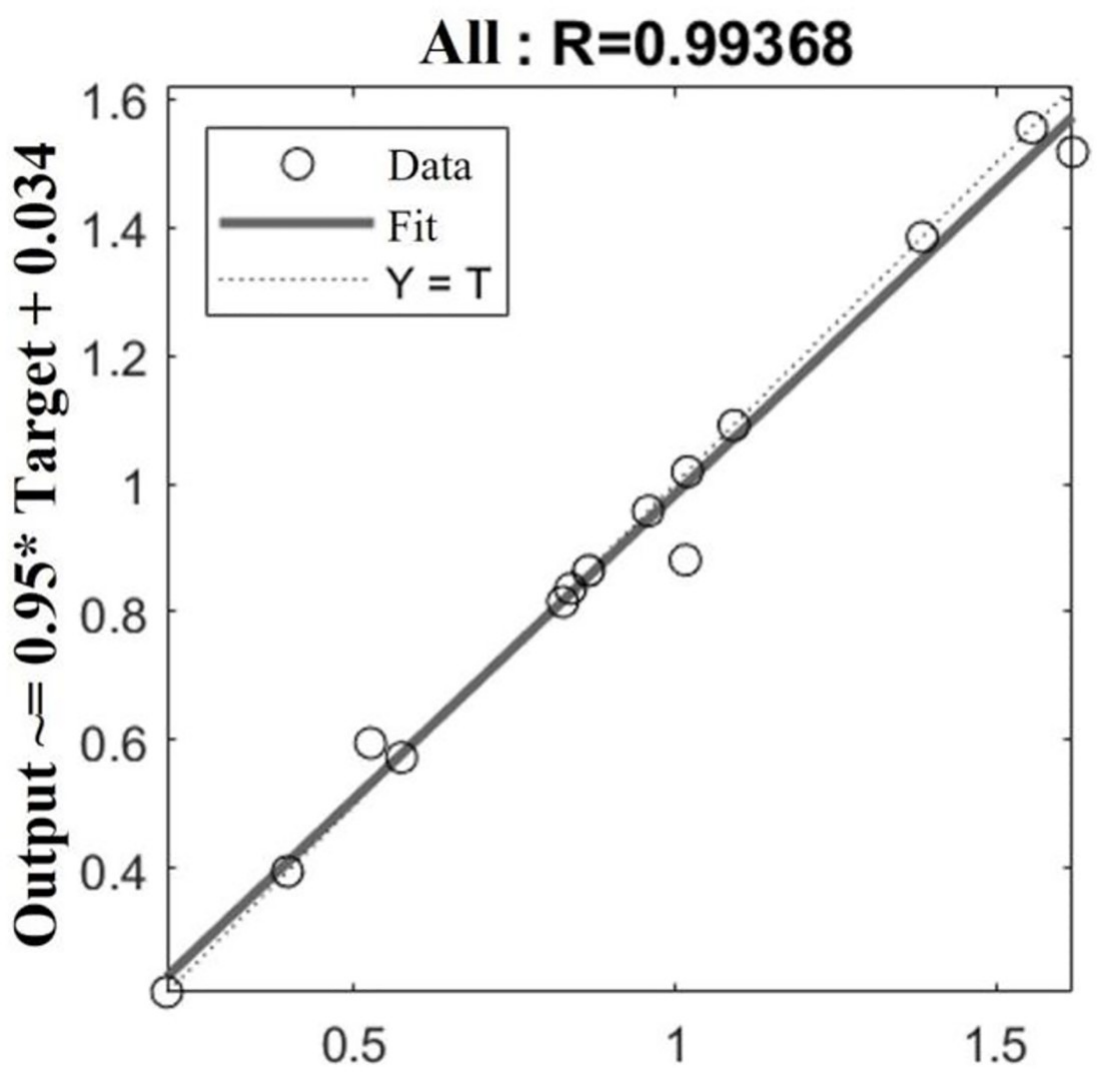
Linear regression analysis diagram.

Fig 13 shows the linear regression between the optimized network and the input value. The correlation coefficient *R* is 0.9936, and the limit is close to 1, proving that the accuracy of the model optimized by the AO algorithm has been significantly improved. It also validates the feasibility of constructing an approximate model using the BP neural network.

## 5 Elevator block brake structural optimization and results

### 5.1 Mathematical model for structural optimization

Structural optimization belongs to computer-aided engineering technology, and its essence is to solve the extreme value problem. Structural optimization is based on mechanical theory and mathematical models, combined with computer technology and a design variable optimization process. Therefore, establishing a correct optimization mathematical model is key to effective structural optimization design. The block brake structural optimization process is equivalent to solving the design variables using Eq 8 as follows [24]:

$$\text{X}={\left[x_{1},x_{1}\cdot \cdot \cdot x_{n}\right]}^{\text{T}}$$ (8)

The objective function under the solution domain of design variables and corresponding constraints is expressed using Eq 9 as follows:

$$s.t.\left\{\begin{array}{l} h\left(\text{X}\right)\leq 0\\ g\left(\text{X}\right)=0 \end{array}\right.$$ (9)

Next, the extreme values are obtained using Eq 10 as follows:

$$f\left(x\right)\to \text{min}\;或\;f\left(x\right)\to \text{max}$$ (10)

where *g*(X) and *h*(X) are the equality and inequality constraints, respectively, and *D* is the design variable solution domain. The design variables can be expressed using Eq 11 as follows:

$$\text{X}={\left[x_{1},x_{2},x_{3}\right]}^{\text{T}}$$ (11)

where *x*_*1*_ is the brake wheel’s outer radius, *x*_*2*_ is the brake shoe width, and *x*_*3*_ is the brake shoe encapsulation angle. This study focuses on bi-objective optimization, and the objective function can be expressed using Eq 12 as follows:

$$f_{obj}=\left\{\begin{array}{l} \text{max}\;N\left(X\right)\\ \text{min}\;M\left(X\right) \end{array}\right.$$ (12)

where *N* (*X*) is the thermal fatigue service life of the brake wheel and *M* (*X*) is the brake wheel mass.

According to the Manson-Coffin formula:

$$\Delta \varepsilon =\Delta \varepsilon_{e}+\Delta \varepsilon_{d}=\frac{\sigma_{f}\prime }{E}{\left(2N_{f}\right)}^{b}+\varepsilon_{f}\prime {\left(2N_{f}\right)}^{c}$$ (13)

where *σ*_*f*_*′* is the fatigue strength coefficient, *N*_*f*_ is the number of stress cycles when fatigue failure occurs, *ε*_*f*_*′* is the fatigue ductility coefficient, *b* is the fatigue strength index, *c* is the fatigue ductility index, and the brake wheel material is HT250. The low-cycle fatigue performance of HT250 was analyzed, and the strain-life relationship formula for this material was obtained using a fatigue prototype test. Eq 13 can be obtained by substituting the material parameters of HT250 into Eq 13.

$$\Delta \varepsilon =\frac{241}{95000}{\left(2N_{f}\right)}^{-0.115}+0.008{\left(2N_{f}\right)}^{-0.36}$$ (14)

Where Δ*ε* is the brake wheel’s total strain amplitude, whose value is related to the brake wheel’s maximum stress and the elastic modulus of the material during emergency braking. *M* (*X*) represents the brake mass in Eq 12, which can be calculated based on its structural characteristics using Eq 15 as follows:

$$M\left(X\right)=\rho \left[\pi \left(x_{1}^{2}-{\left(x_{1}-0.02\right)}^{2}\right)\right]x_{2}$$ (15)

Accordingly, this study includes two inequality constraints, which can be expressed using Eq 16 as follows:

$$s.t.\left\{\begin{array}{l} T\left(\text{X}\right)\leq 300\\ S\left(\text{X}\right)\leq 250 \end{array}\right.$$ (16)

where *T* (*X*) is the highest temperature of the brake wheel friction surface during emergency braking, and its value should be less than 300°C; *S* (*X*) is the maximum thermal stress generated by the brake wheel during emergency braking, and its value should be less than the brake wheel’s material yield stress. In summary, the mathematical model for the optimization problem can be expressed using Eq 17 as follows:

$$\left\{\begin{array}{l} \;\text{max}\;N\left(X\right)\\ \;\text{min}\;M\left(X\right)\\ 0.55\leq x_{1}\leq 0.65;.0.10\leq x_{2}\leq 0.14\\ 17\leq \theta \leq 19\\ s.t.T\left(X\right)\leq 300;S\left(X\right)\leq 250 \end{array}\right.$$ (17)

### 5.2 Optimization algorithm determination

The structural optimization design of a block brake is a bi-objective optimization problem, and generally, there are two approaches to solving this. One is to convert a multi-objective problem into a single-objective solution. The other is to use an intelligent optimization algorithm to obtain the Pareto optimal solution [25]. This study determined the optimal mathematical model design for a block brake by adopting a multi-objective genetic algorithm. The multi-objective genetic algorithm adopts fast, non-dominated sorting and introduces multiple target operators based on the GA [26]. Compared with the single-objective genetic algorithm, the crossover and mutation methods remain unchanged, and the individual selection method is improved. Compared with traditional multi-objective algorithms, the multi-objective genetic algorithm demonstrates an improved processing ability for nonlinear problems under multidimensional datasets and multi-constraint conditions.

In this study, the multi-objective optimization problem can be expressed by Eq 18 as follows:

$$\text{min}\;\left[f_{1}\left(x\right),f_{2}\left(x\right),\cdot \cdot \cdot ,f_{m}\left(x\right)\right];s.t.\left\{\begin{array}{l} l_{b}\leq x\leq ub\\ Aeq*x=beq\\ A*x\leq b \end{array}\right.$$ (18)

where *lb* and *ub* represent the upper and lower limits of the design variable *x*, Aeq* *x* = *beq* represents the optimization problem equality constraint, and A* *x* ≤*b* represents the optimization problem inequality constraint.

The upper and lower limits of design variable *x* are as follows:

$$lb=\left[0.\text{55},0.\text{1}0,\text{17}\right],\;ub=\left[0.\text{65},0.\text{14},\text{19}\right]$$ (19)

In addition, when a multi-objective genetic algorithm is adopted to solve the objective function extreme value problem, the minimum or maximum values of the two objective functions must be considered. As shown in Eq 20, the following transformation is conducted:

$$\text{max}\;N\left(X\right)\to \text{min}\;\left(-N\left(X\right)\right)$$ (20)

### 5.3 Optimization process and results

**Fig 14:**
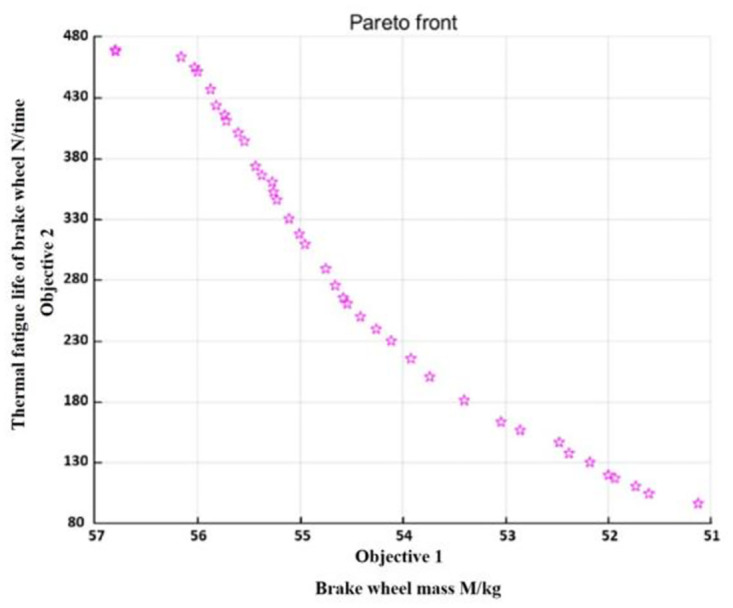
Pareto optimal solution.

For the optimization problem, the two objective functions were contradictory. If one objective function must be improved, the other must be reduced. As shown in Fig 14, objective 1 or 2 is typically a non-inferior solution with multi-objective optimization, also known as the Pareto optimal solution.

This study adopts the multi-objective optimization method based on the GA to optimize the ventilated disc brake. First, the multi-objective optimization GA’s population size is set at 100, the maximum genetic algebra is 500, and the optimal front-end individual coefficient is 0.4. The product of the optimal front-end individual coefficient and population size is the number of Pareto optimal solutions that must be output. Therefore, 0.4 × 100 = 40 Pareto optimal solutions should be output for this study. The Pareto optimal solutions are shown in Table 4.

**Table 4:** The approximate model outputs Pareto optimal solutions.

Pareto solutions	*x*_1_	*x*_2_	*x*_3_	M	N
1	0.5856	0.1003	18.91	52.27	114
2	0.5809	0.1003	17.07	51.84	96
3	0.6364	0.1003	17.01	56.88	463
4	0.5824	0.1003	18.85	51.97	96
5	0.5896	0.1003	17.28	52.63	137
6	0.5908	0.1002	18.93	52.74	145
7	0.5936	0.1002	18.91	52.99	166
8	0.5739	0.1002	18.92	51.61	93
9	0.5912	0.1002	18.93	52.77	147
10	0.5942	0.1003	18.94	53.05	173
11	0.5870	0.1002	18.88	52.40	120
12	0.5974	0.1002	18.92	53.34	199
13	0.5841	0.1003	18.85	52.13	106
14	0.6215	0.1002	18.53	55.52	368
15	0.6018	0.1002	18.81	53.74	232
16	0.6076	0.1002	18.72	54.26	283
17	0.5889	0.1002	18.94	52.57	132
18	0.6248	0.1002	17.12	55.82	397
19	0.6364	0.1002	17.01	56.88	476
20	0.6152	0.1002	18.31	54.96	318
21	0.5847	0.1002	18.95	52.18	110
22	0.6001	0.1002	18.92	53.59	222
23	0.5987	0.1002	18.96	53.46	213
24	0.5925	0.1002	18.93	52.89	158
25	0.6267	0.1002	17.13	56.00	425
26	0.5964	0.1002	18.87	53.25	188
27	0.5983	0.1003	18.95	53.42	208
28	0.6297	0.1003	17.45	56.27	449
29	0.6274	0.1003	17.58	56.07	431
30	0.5857	0.1003	18.95	52.28	116
31	0.5877	0.1002	18.86	52.46	123
32	0.6173	0.1002	18.50	55.14	351
33	0.6034	0.1002	18.72	53.89	242
34	0.5827	0.1002	18.8563	52.02	100
35	0.6225	0.1002	18.2231	55.62	387
36	0.6113	0.1002	18.6335	54.60	311
37	0.6054	0.1002	18.6965	54.08	259
38	0.6311	0.1002	17.1613	56.39	464
39	0.5907	0.1002	18.8299	52.73	137
40	0.5739	0.1003	17.0214	51.20	93

Because the Pareto optimal solution of multi-objective optimization cannot achieve the optimal solution of all objectives simultaneously, when one of the optimization efforts is strengthened, the optimization efforts of other objectives must be weakened. Therefore, in the output of 40 groups of Pareto optimal solutions, the number of cyclic stresses is appropriately increased while considering reducing the quality objective as much as possible. After a comprehensive analysis, the 11th data group was selected as the optimized result. Table 5 shows that *T*_max_ and *S*_*max*_ were obtained by substituting the Pareto solution into the approximate model.

**Table 5:** Pareto optimal solution finite element simulation result.

Variable	*x*_1_	*x*_2_	*x*_3_	*T*_max_	*S*_max_	*M*	*N*
Result	0.5870	0.1002	18.88	212.01	234.24	52.40	120

**Fig 15:**
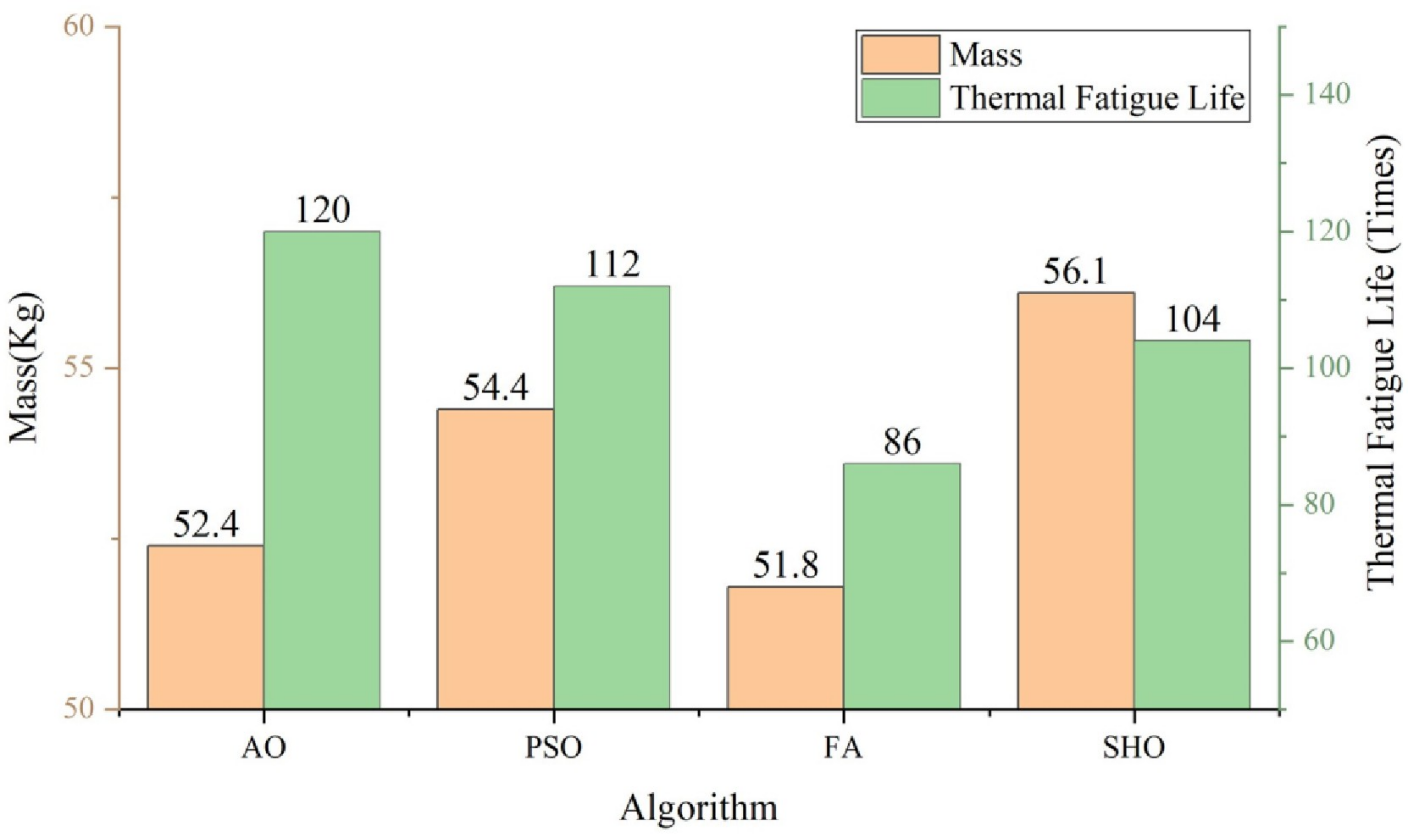
Optimization results of different algorithms.

In order to confirm the effectiveness and superiority of the proposed method, the author compares the optimization results of several different algorithms in the dual-objective structure optimization problem of the brake wheel. As shown in Fig 15, the thermal fatigue life optimization effect of the AO algorithm on the brake wheel is higher than that of the other three algorithms, and the quality optimization result of the brake wheel is only slightly worse than that of the FA algorithm, although it is not optimal. However, the optimization result for thermal fatigue life is much higher. In general, the approximate physical model method based on the AO algorithm in this study is reliable and effective in dealing with the structural optimization of brake wheels.

### 5.4 Optimization result analysis and verification

Table 6 compares the design variables and results before and after optimization. As observed, in addition to the increased brake shoe encapsulation angle, other structural parameters were reduced, among which the brake wheel diameter was reduced from 600 to 587 mm. The brake shoe width was reduced from 120 to 100.2 mm. The brake shoe encapsulation angle varied from 18 to 18.87°. In addition, the maximum temperature of the brake wheel output by the approximate model is reduced by 18% from 258.8°C to 212.01°C, and the maximum stress is also reduced by 15% from 275.66 MPa to 234.24 MPa. The final optimized brake wheel mass is reduced from the original 58.85 kg to 52.40 kg, a reduction of 11%, and the thermal fatigue life of the brake wheel is increased from the original 59 times to 120 times, an increase of 103%.

**Table 6:** Comparison of parameters before and after optimization of structural parameters.

	Brake wheel diameter (*x*_1_/mm)	Brake shoe width (*x*_2_/mm)	Brake shoe encapsulation angle (*x*_3_/mm)	Temperature (*T*/°C)	Stress (*S*/MPa)	*M* (kg)	*N* (Time)
Before optimization	600	120	18°	258.8	275.66	58.85	59
After optimization	587	100.2	18.87°	212.01	234.24	52.40	120
Rate of change	2.16%	16.5%	5%	18%	15%	11%	103%

**Fig 16:**
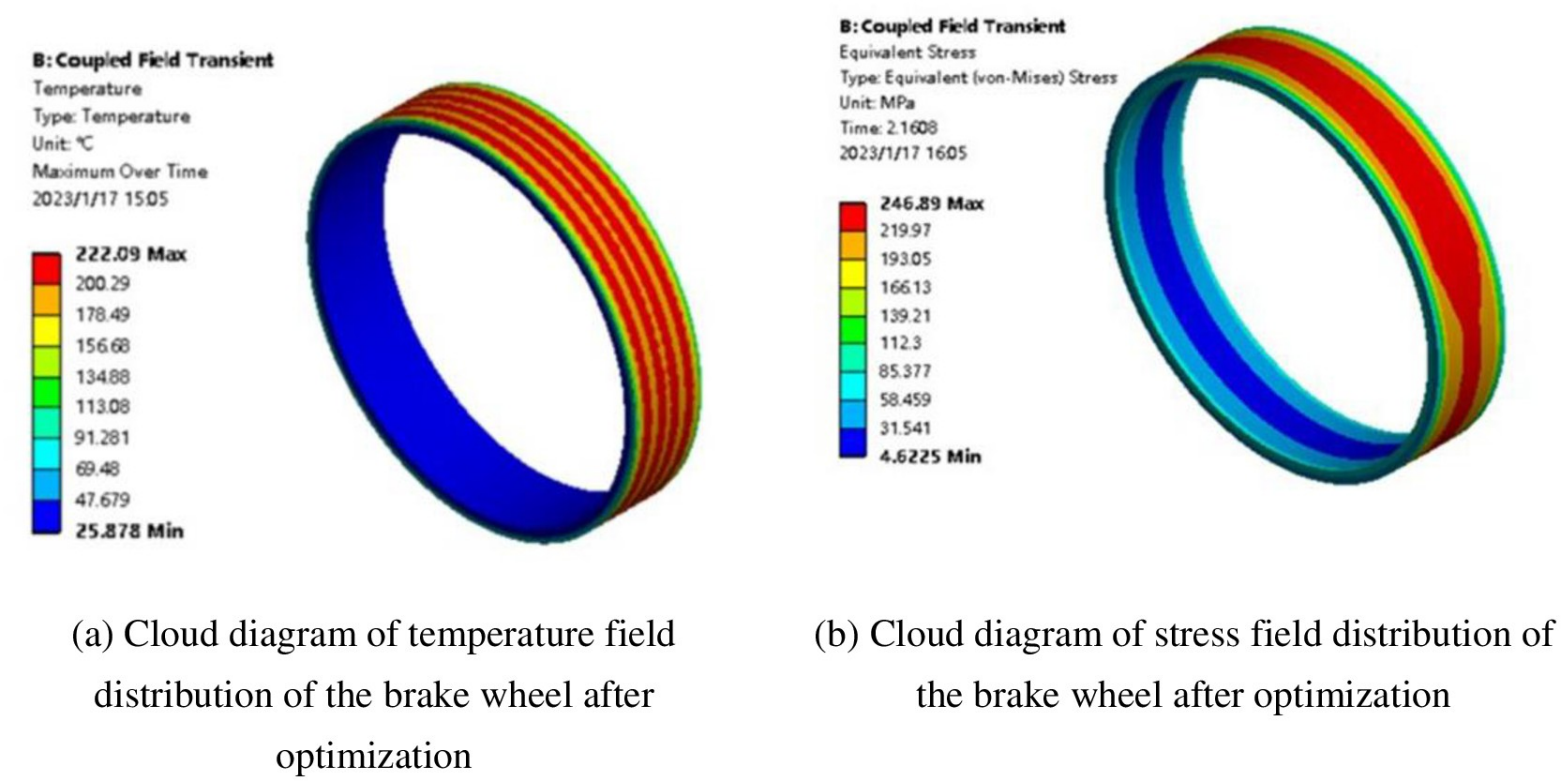
Cloud diagram of finite element simulation analysis of an optimized brake wheel. (a) Cloud diagram of temperature field distribution of the brake wheel after optimization, (b) Cloud diagram of stress field distribution of the brake wheel after optimization.

Some errors may exist because the optimization results were obtained by substituting the optimized design variables into the established approximate model. To further verify the accuracy of the optimization results, the thermal-structural coupling finite element model for the elevator block brake was re-established according to the optimized design variables. Simultaneously, identical boundary conditions and loads were applied for the simulation calculations, and the approximate model output value was verified. Fig 16(a) and 16(b) show the temperature field distribution cloud diagram corresponding to the brake wheel’s maximum temperature and the stress field distribution cloud diagram corresponding to the maximum stress moment during the emergency braking process, respectively. According to Fig 14, the actual maximum temperature of the brake wheel was 222.09°C, 10.08°C higher than the brake wheel maximum temperature output determined by the approximate model of 212.01°C. The maximum equivalent stress of the actual brake wheel was 246.89 MPa, 12.65 MPa higher than the maximum stress of the 234.24 MPa output determined by the approximate model. The thermal fatigue life of 246.89 MPa was solved by Eq 11 94 times; that is, it increased from 64 to 94 times after optimization, proving that the approximate model established by the proposed AO-BP neural network is highly accurate. Fig 16 shows the cloud diagram of finite element simulation analysis of an optimized brake wheel.

## 6 Conclusion

The results of this study indicate that the proposed method can be effectively used to optimize the block brake structure of an elevator based on an AO-BP neural network. The conclusions of the proposed method are summarized as follows:

1. The maximum temperature of the optimized brake wheel was significantly lower, with a decrease of 14.2%.
2. The optimized brake wheel maximum equivalent stress was reduced by 10.5% compared with that before optimization.
3. A lightweight brake wheel mass design was achieved, which was reduced from 58.85 to 52.40 kg after optimization.
4. The thermal fatigue life at the brake wheel maximum equivalent stress increased significantly from 64 to 94 times after optimization, proving that the proposed solution effectively improves the structural parameters and performance of elevator brakes.

The emergency braking process of an elevator brake is a mutual coupling of multiple physical fields. Although some results are obtained in the block brake structure optimization of the elevator, some imperfections should be improved. Next, if experimental conditions permit, a brake prototype can be produced based on the optimized quality, with testing to verify the effectiveness of the optimized parameters and the credibility of thermal fatigue life obtained from simulation analysis. In addition, the temperature sensors can be installed inside the brake shoes to detect the real-time temperature data of the brake shoes during the emergency braking experiments, thereby verifying the accuracy of the finite element model’s simulation results.
